# Dynamics of native oxide growth on CdTe and CdZnTe X-ray and gamma-ray detectors

**DOI:** 10.1080/14686996.2016.1250105

**Published:** 2016-11-21

**Authors:** Jakub Zázvorka, Jan Franc, Lukáš Beran, Pavel Moravec, Jakub Pekárek, Martin Veis

**Affiliations:** ^a^ Faculty of Mathematics and Physics, Institute of Physics, Charles University in Prague, Prague, Czech Republic

**Keywords:** CdTe, oxidation, ellipsometry, XPS, leakage current, 40 Optical, magnetic and electronic device materials, 212 Surface and interfaces, 201 Electronics / Semiconductor / TCOs

## Abstract

We studied the growth of the surface oxide layer on four different CdTe and CdZnTe X-ray and gamma-ray detector-grade samples using spectroscopic ellipsometry. We observed gradual oxidization of CdTe and CdZnTe after chemical etching in bromine solutions. From X-ray photoelectron spectroscopy measurements, we found that the oxide consists only of oxygen bound to tellurium. We applied a refined theoretical model of the surface layer to evaluate the spectroscopic ellipsometry measurements. In this way we studied the dynamics and growth rate of the oxide layer within a month after chemical etching of the samples. We observed two phases in the evolution of the oxide layer on all studied samples. A rapid growth was visible within five days after the chemical treatment followed by semi-saturation and a decrease in the growth rate after the first week. After one month all the samples showed an oxide layer about 3 nm thick. The oxide thickness was correlated with leakage current degradation with time after surface preparation.

## Graphical abstract

**Figure:**
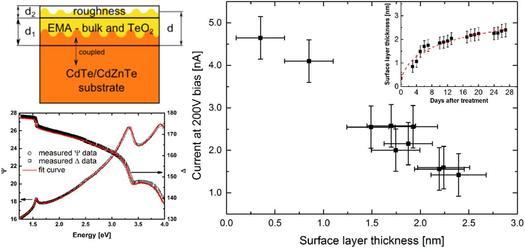

## 1. Introduction

In the last decade significant scientific interest has been paid to the materials CdTe and CdZnTe due to their applications as room-temperature spectroscopic X-ray and gamma-ray semiconductor detectors. They have a relative large bandgap at room temperature, E_g_ ~1.5–1.6 eV and a high average atomic number. They can be prepared as semi-insulating materials with high resistivity ~1·10^10^ Ωcm, which is necessary to achieve a good signal-to-noise ratio of the radiation detectors. Lately, it has been shown that the surface treatment and its effects on the metal/semiconductor interface and lateral sides can significantly influence the detector performance.[1–5] The impact of mechanical polishing and chemical etching in Br-methanol solutions has been studied by measurements of I–V characteristics and photocurrent,[6–9] while the surface morphology was investigated using optical microscopy, interferometry and X-ray photoemission spectroscopy (XPS) measurements.[10–12] However, the published results did not present a clear conclusion about an optimal surface treatment process from the detector performance viewpoint. Moreover, time evolution of the leakage current and of the detector quality was observed.[12,13]. This has been attributed to oxidization of the detector surface. Even without any passivation a thin layer of oxide grows on the CdTe surface when exposed to ambient air. Previously published XPS results suggest that the oxygen is almost exclusively bound to tellurium,[12,14], forming a layer consisting of mainly TeO_2_ and CdTeO_3_. Several studies have been made employing spectroscopic ellipsometry to evaluate the surface layer thickness in dependence on the preparation techniques used and on the oxide atomic ratio obtained by XPS measurements.[15–17] However, a systematic study of the dynamics of native oxide formation and the oxide layer thickness evolution with respect to the time after the surface treatment has not been reported yet. The knowledge of these dynamics may help to better understand the process of the surface oxide formation, which is crucial for the development of a suitable surface treatment technique and to ensure a long time functional stability of the detectors. In our previous work [18] we have studied the influence of CdZnTe surface treatment on resistivity and photoconductivity measurement and evaluated the surface oxide layer thickness using spectroscopic ellipsometry and XPS measurements within a day after the surface treatment. In this paper, we present a systematic study of the evolution of the surface oxide layer thickness on various CdTe and CdZnTe samples, prepared by different growth methods and originating from several suppliers, with respect to the time (up to 30 days) after the sample treatment. The goal of the research was to evaluate the oxide growth rate and its effect on the leakage current. Achieving the lowest possible leakage current is crucial since it correlates with the spectral resolution of the detector.[19,20] Considering this, the surface oxide layer seems to be another vital parameter in fabrication of CdTe and CdZnTe X-ray and gamma-ray detectors.

## 2. Experimental procedures

We studied four different samples, three from commercial suppliers (Samples 1–3) and one sample grown at the Institute of Physics at Charles University in Prague using the vertical-gradient-freeze (VGF) method (Sample 4). Sample parameters, including dimensions and their average dark resistivity (determined by the contactless resistivity measurement) are shown in Table 1.

**Table 1.** Sample designation and parameters.

Designation	Material	Doping	Zn concentration (%)	Dimensions (mm^3^)	Resistivity (Ωcm)
Sample 1	CdTe	Cl	0	4 × 4 × 1.5	~4·10^9^
Sample 2	CdZnTe	In	~10	5 × 5 × 2	~1·10^10^
Sample 3	CdZnTe	In	~10	7 × 4 × 2	~2·10^10^
Sample 4	CdTe	In	0	6 × 6 × 2	~9·10^8^

All samples, except for Sample 3, had no contacts. The side with the largest surface area was used in the measurements. All samples were chemo-mechanically polished on a silk pad using a 3% Br-ethylene glycol solution for 60 s. Afterwards they were chemically etched by immersion into a 3% Br-methanol (Br-MeOH) solution for 60 s. On average 20 μm off the top of each sample were removed after the chemical polishing and etching, which was measured by digital indicator with the resolution of 1 μm. The samples were kept on ambient air at room temperature after the surface treatment and between measurements. XPS spectra were measured immediately after the sample etching and after three weeks of ambient air exposure. The spectra were taken using Al K_α_ X-ray source (hυ = 1486.6 eV) and measured at normal emission angle by 16 channel HA-100 hemispherical electron analyzer supplied by VSW in an ultrahigh vacuum chamber with a base pressure below 1 × 10^−7^ Pa.

We have studied the optical response of the samples by spectroscopic ellipsometry with respect to the time after etching. Ellipsometry is a non-destructive optical technique, which proved itself as an effective tool for derivation of optical properties of matter and surface layer thickness.[21] It measures changes in light polarization upon reflection from the sample. This polarization change is represented by ellipsometric angles Ψ and Δ, which are related to Fresnel reflection coefficients for s- and p-polarized light by:

(1) $$\rho =\frac{r_{p}}{r_{s}}=\tan\left(\Psi \right)\cdot e^{i\Delta }$$

The measured parameters Ψ and Δ are sensitive to surface conditions, layers thicknesses and dielectric functions of investigated materials. Therefore, with proper choice of a theoretical model structure one can fit the experimental data and derive spectrally dependent optical properties of investigated material as well as the thickness of the surface oxide layer. In our case the model structure consisted of a semi-infinite bulk CdTe material with the surface oxide layer of certain thickness and roughness. An assumption of semi-infinite CdTe is justifiable owing to its high absorption coefficient in the investigated spectral region and a large thickness of the sample with respect to the surface layer. A commercial Mueller matrix ellipsometer RC2 (J.A. Woollam Co., USA) was used to obtain the experimental data in the spectral range from 1.2 to 4 eV. The results were fitted with a theoretical model and the mean squared error (MSE) was used to evaluate the reliability of the fit. Sample 3 was selected for electrical I–V measurements and their correlation with the oxide evolution. Gold contacts were chemically deposited at the 4 × 2 mm^2^ lateral sides of this sample by immersion into aqueous AuCl_3_ solution for 1 min. The sample was biased using the Keithley 2410 Sourcemeter (Tektronix Inc., USA) and the current was measured on a serial 100 Ω resistor using the Keithley 6514 Electrometer.

## 3. Results and discussion

**Figure 1.**
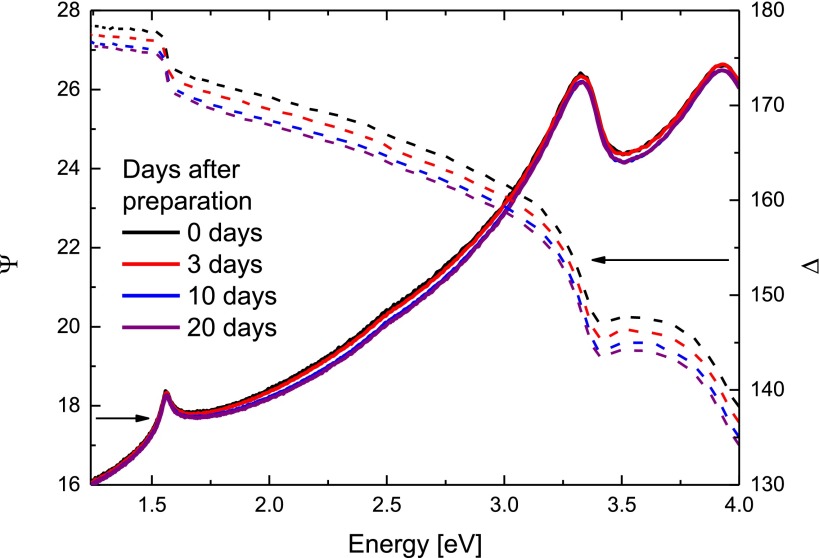
Evolution of ellipsometric parameters *ψ* (solid lines) and Δ (dashed lines) for Sample 3 after the surface treatment. The data were acquired at incident angle of 60°.

Ellipsometry was measured in reflection for three incident angles Φ = 55°, 60° and 65°. The use of variable-angle spectroscopic ellipsometry (VASE) was necessary to determine the surface layer thickness with good accuracy. Figure 1 shows a typical evolution of parameters Ψ and Δ with time after the surface treatment. The incident light spot diameter was 4 mm. In this case, most of the sample was illuminated and the gathered information was averaged over the whole surface. From Figure 1 one can see a significant impact of time on the spectra of the Δ parameter, which is related to absorption of the sample. The variation of the parameters with time after etching suggests certain changes at the surface.

**Figure 2.**
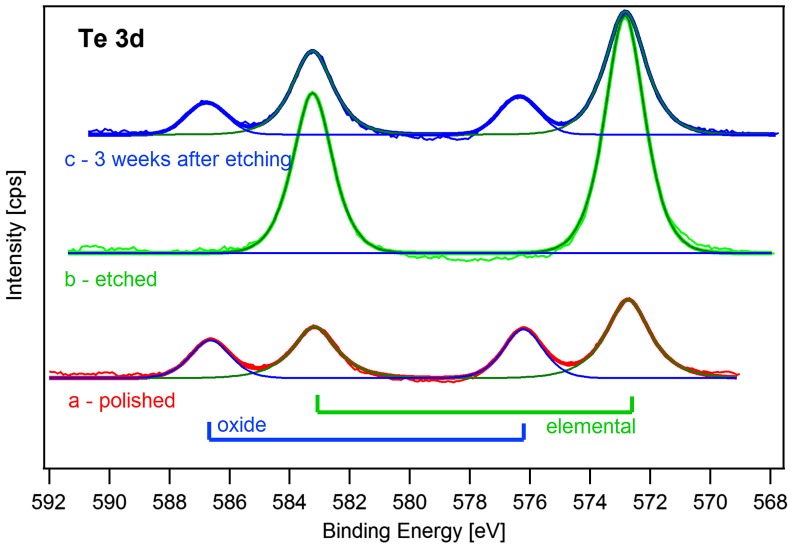
(a) XPS spectra for mechanically polished surface of Sample 3, (b) surface etched with Br-MeOH for Sample 3 within an hour after the preparation and (c) Sample 3 three weeks after chemical etching. The ratio of oxygen bound on tellurium to elemental tellurium is (a) 0.55, (b) 0.02 and (c) 0.30, showing that the damaged layer after the mechanical polishing which contains oxygen can be removed by etching in Br-MeOH; three weeks after etching the oxide layer bound to tellurium is re-established.

To justify the assumption of the oxide layer growth XPS was measured on Sample 3 and the results are shown in Figure 2. Te3d spectra were measured after the initial preparation by mechanical polishing – red line in Figure 2. A peak doublet of elemental tellurium is visible at energies 573 eV and 583 eV, marked as ‘elemental’. Another peak doublet related to the oxygen bound to tellurium is also visible in Figure 2, marked as ‘oxide’. Because the doublet shift is about 3.3 eV, the measurement indicates a formation of the TeO_2_ layer [16, 22]. Then the sample was chemically etched and we performed the XPS experiment again within an hour after the etching. The XPS spectrum shows no signal of oxygen bound to tellurium – green line in Figure 2. After three weeks of exposure of the sample to ambient air we measured XPS again (see Figure 2, line c). The spectra showed oxidization of the sample. The XPS measurements revealed no Cd- or Zn-bound oxide peaks immediately after etching and after three weeks. Therefore, we conclude that there is no CdO or ZnO formation at the surface. However, the peaks of oxygen bound to tellurium were clearly visible. This way we confirmed a formation of a TeO_2_ surface layer after keeping the sample three weeks on ambient air at room temperature. The thickness of the oxide layer can be evaluated as described in [17]. After chemical etching, no oxide is detected by XPS. Within three weeks a layer of approx. 1.5 nm oxide is formed, when evaluating the XPS signal. However, the commonly used theory for an oxide layer thickness determination using XPS supposes an extremely flat surface and requires to determine the attenuation length of the XPS (for this measurement taken from [17]). After chemical etching, CdTe surface exhibits a greater roughness as compared to mechanical polishing.[11] The morphology unevenness is greater than the attenuation length of XPS and another method must be implemented for more accurate thickness measurements.

**Figure 3.**
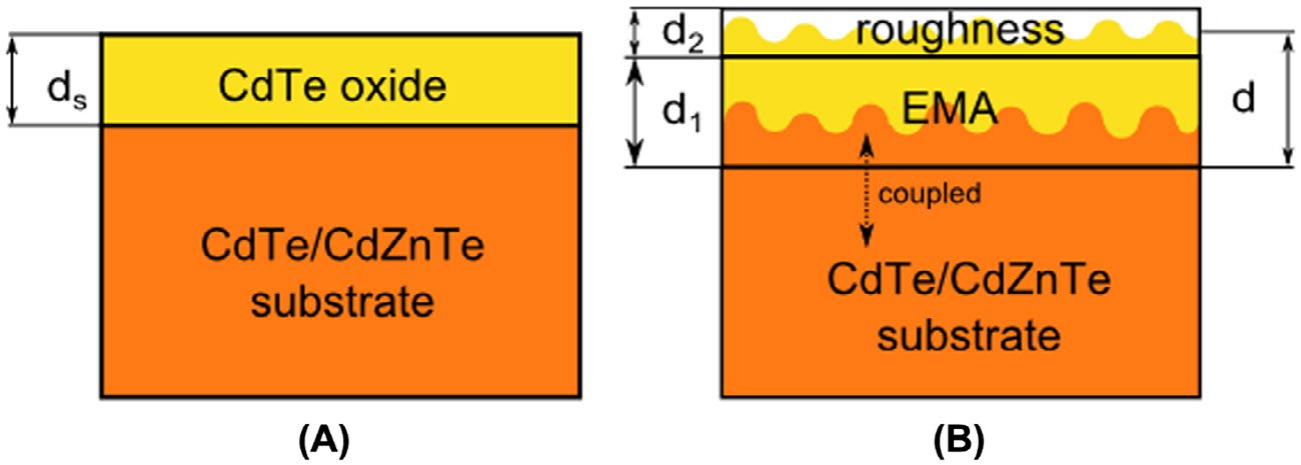
(A) simple model with flat surface (not used in our evaluation); (B) model used for etched samples with an effective-medium-approximation (EMA): coupled substrate and CdTe oxide.

The growth of the surface oxide layer and correlation between metallic tellurium layer evaluated with ellipsometry and tellurium peak height and width in XPS was proven already by Badano et al. [16]. However, a detailed insight into the dynamics and the oxide growth rate after the chemical preparation has not been published yet. Our work concentrates on the evaluation of the oxide layer thickness in time after etching. It aims to determine the impact of air exposure of the CdTe and CdZnTe samples during the fabrication process on the thickness of the surface oxide layer. To evaluate the thickness of the oxide layer we had to compare the experimental data with a theoretical model of the surface structure. Yao et al. [15] investigated mechanically polished samples and used a simple layer of CdTe–oxide and an intermix of CdTe–oxide and void on a CdTe substrate. Badano et al. [16] used a more complicated structure consisting of a substrate, intermix layer, metallic tellurium layer, oxide layer and surface roughness. Because the etching process induces a higher roughness of the surface, the simple model by Yao et al. [15] seems insufficient for ellipsometry investigation. Therefore we have focused on the next simplest model that involves a mix of both approaches and proposed a model with three components – the semi-infinite CdTe bulk, layer consisting of bulk CdTe and oxide layer, described by effective-medium-approximation (EMA), and surface roughness layer. The scheme of the model is shown in Figure 3.

In EMA the optical parameters of the consisting materials are mixed with the ratio from 0 to 100%. This approach practically substitutes a non-uniform surface layer with material peaks and trenches. Moreover, the optical parameters of bulk CdTe material were coupled to the parameters used in the EMA layer. The idea was that the CdTe bulk itself has a non-uniform surface and the oxide layer grows upon its roughness. The angular spread on the sample surface was also taken into account.

**Figure 4.**
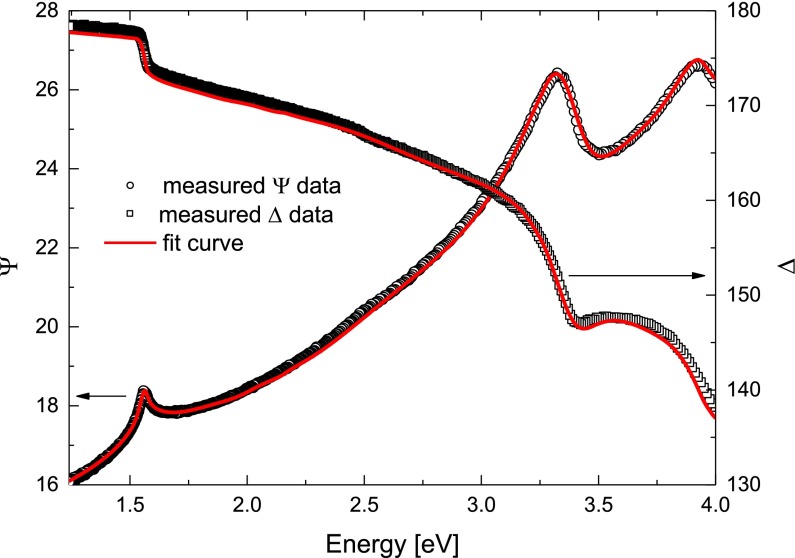
Representative plot of the fitting of measured data. The scatter points represent the data measured on Sample 3 right after surface preparation. The solid red lines demonstrate the fit agreement with the measurement. Selected dataset had the highest MSE, remaining fits showed even better fit reliability.

To fit the experimental data, CompleteEASE software provided by J.A.Woollam Co., USA was used. The initial optical parameters of bulk CdTe and parameters of the CdTe oxide were taken from the database supplied by Woollam Co. Inc (USA). The CdTe bulk was parametrized using Lorentz oscillators and the CdTe oxide was parametrized with the Cauchy approximation. We analyzed the experimental data using multi-sample analysis (MSA). In our case the analysis consisted of multiple measurements performed on each sample with respect to the time after the surface treatment. The optical parameters of the bulk and of the CdTe oxide were set the same for the whole data ensemble. Bulk parameters were fitted because of expected variations in absorption edges across the samples, the parametrized optical parameters of CdTe oxide were taken from the database and remained unchanged during fitting. Only the composition ratio of the EMA, the thickness of the EMA layer *d*_1_ and the roughness *d*_2_ could change individually in each measurement. In Figure 4 the scatter points show measured data of Sample 3 and the solid lines represent the fit using the model structure described above.

**Figure 5.**
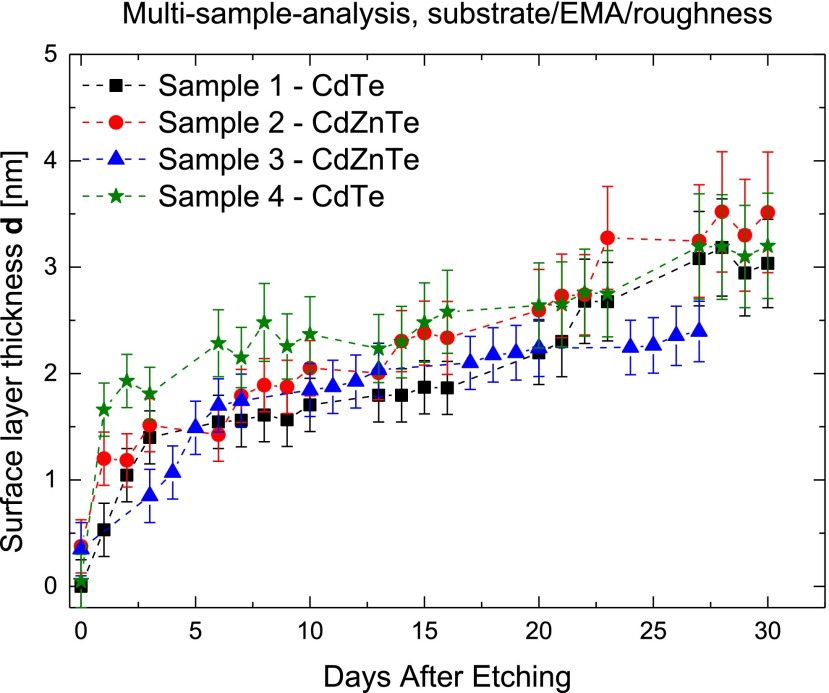
Evolution of surface layer thickness **d** after the surface preparation treatment. Lines serve as a guide to the eye.

Because of the interface roughness between the bulk and the oxide layer, we evaluated the overall surface layer thickness as the thickness of the EMA layer *d*_1_ and a half of the roughness *d*_2_ (*d* = *d*_1_+0.5 × *d*_2_), as shown schematically in Figure 3(b). The time evolution of the surface layer thicknesses for all samples is shown in Figure 5.

CdTe samples 1 and 4 exhibit almost no surface layer right after the surface treatment, whereas CdZnTe samples 2 and 3 show a surface layer with the thickness approx. 0.5 nm right after the surface etching. Interestingly, these are the CdZnTe samples with 10% zinc concentration. As mentioned above, no traces of Zn-bound oxide were found in the XPS spectra. The reason for the presence of the initial oxide right after etching might be a different stoichiometry of the sample in the case of CdZnTe. The content of zinc increases the ionic character of the atom bonds. However, the accuracy of the ellipsometry evaluation could play a vital role in the initial oxide determination. We have estimated the thickness error when investigated by ellipsometry to be 0.5 nm based of mathematical evaluation of mean-square-error of the data fitting.

The growth rate of the surface layer on all samples within one month is visible in Figure 5. The growth of surface oxide on all samples resembles a field assisted oxide growth [23] as it follows the logarithmic law described in [23, 24]. This was already reported by Özsan et al. [17] after etching in a weaker bromine solution. However, no correlation with the leakage current was made. The oxide growth seems to have a semi-saturation in about five days for all of the samples. Within these first five days, a fast oxidation of the surface occurs and is slowed afterwards. Within one month after the chemical treatment, all the samples show about 3 nm thick surface layer. The fit error increases with larger layer thicknesses. This can indicate that the parameters of the theoretical model can vary, e.g. the evaluation program cannot decide whether a layer thickness should increase or the EMA composition (bulk, oxide and void mixtures) of the layer is changing. In our evaluation we kept the oxide layer optical parameters constant in the whole evolution datasets. Even so, the dynamics of the oxide layer growth is clearly visible. We also found that within the first growth phase (up to five days) the evolution of the EMA layer thickness *d*_1_ has the greatest contribution to the surface layer thickness *d*. The ratio of CdTe-oxide in the EMA layer is about 95% during the first five days after the etching. Afterwards, it gradually decreases to 70–80% in all samples. In the slower oxide growth phase, both the EMA layer thickness *d*_1_ and the surface roughness *d*_2_ are increasing similarly. The reason for the increase in roughness might be attributed to mechanical stress and lattice mismatch of the oxide layer, resulting in a non-uniform columnar surface structure. This is also a feature of the field-assisted growth.[23]

**Figure 6.**
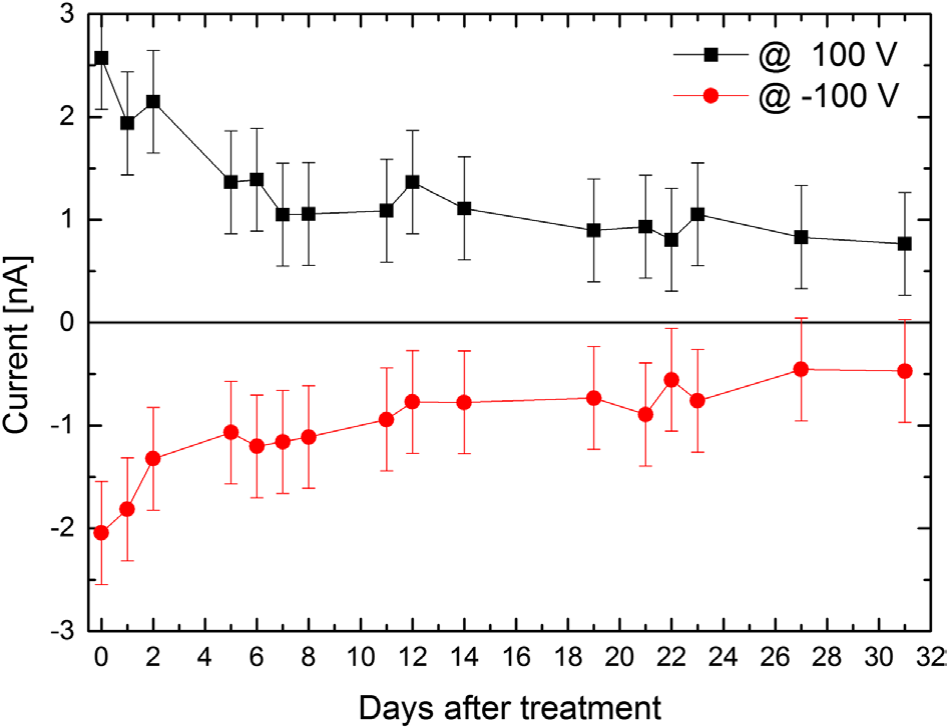
Current evolution of Sample 3 biased at 100 V (both polarities) after the surface treatment. Lines serve as a guide to the eye.

**Figure 7.**
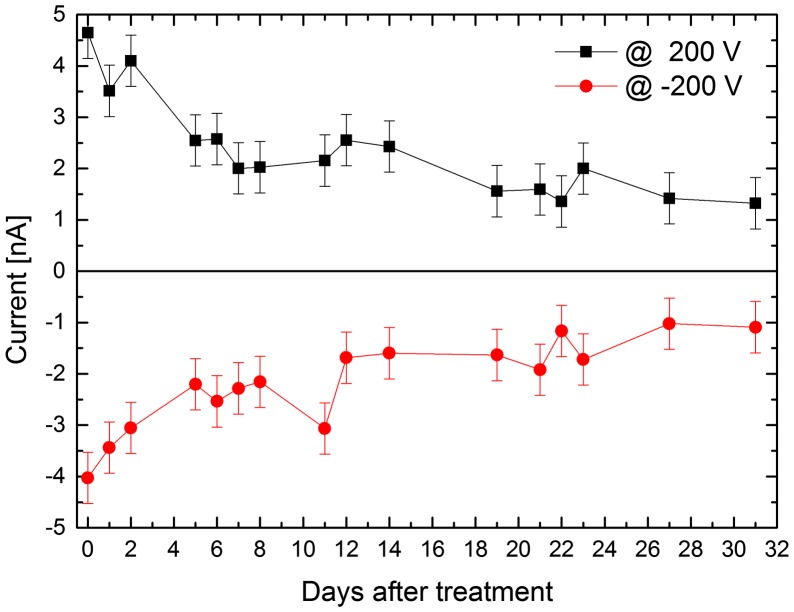
Current evolution of Sample 3 biased at 200 V (both polarities) after the surface treatment. Lines serve as a guide to the eye.

The I–V characteristics of Sample 3 were measured and correlated with the surface layer thickness evolution. Figures 6 and 7 show the evolution of the measured current within 31 days after the surface treatment. We can see that the current through the sample relaxes over the time towards lower values. Also the semi-saturation after about five days is clearly visible. Because the decreased current values correlate with thicker surface layer evaluated from ellipsometry, we conclude that the thicker surface layer influences the detector by passivating of the lateral sides. The reason for the leakage current reduction may be the influence of the oxide on the sample band structure. The oxide thickness (and the charge stored in the surface layer) induces band bending. This results in a depletion layer on the sample surface. While the current flows also in some depth from the surface, the depletion layer reduces its amount by posing as a highly resistive region. The reduced leakage current might have a further influence on the spectral resolution of the final detectors. As the oxide thickness changes in time after the etching and correlates with leakage current, we think it is a necessary parameter in the CdTe and CdZnTe radiation detector development.

**Figure 8.**
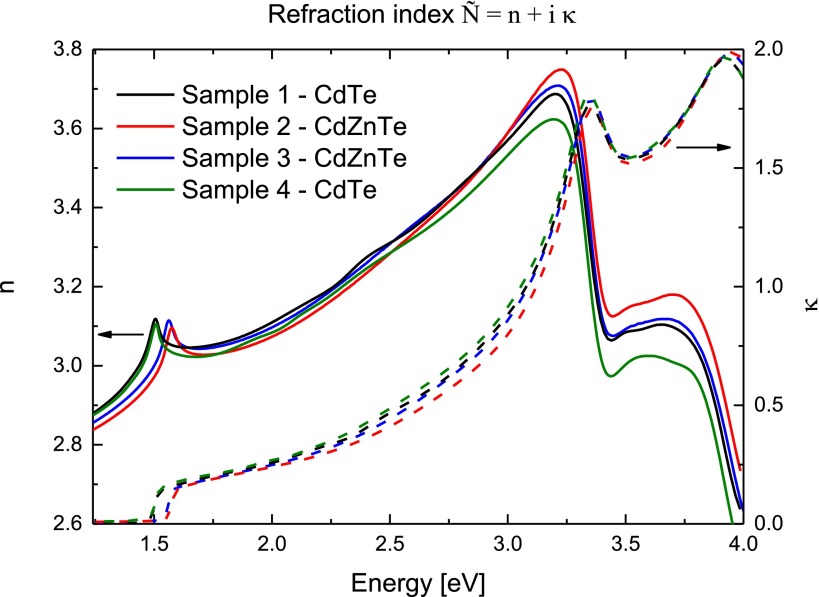
Optical parameters of bulk parts of Samples 1–4 determined by VASE.

While determining the surface layer thicknesses, we also evaluated the optical parameters of the bulk CdTe and CdZnTe, as shown Figure 8.

From Figure 8 we can see that the absorption edge moves to higher energies in the samples with higher zinc concentrations. This is in agreement with theoretical predictions of the CdTe and CdZnTe bandgap energies and another measurements,[25] and indicates the correctness of the data fitting.

## 4. Conclusions

We have investigated two CdTe and two CdZnTe samples from different suppliers using the variable-angle-spectroscopic-ellipsometry and complementary XPS measurements. As a starting treatment we used chemical etching by immersion into 3% Br-methanol solution. The changes in the ellipsometry parameters Ψ and Δ were studied with respect to the time of air exposition at room temperature. We proposed a refined theoretical model of the sample surface structure and then estimated the time evolution of the surface layer thickness of CdTe and CdZnTe. In CdTe samples no initial oxide layer right after etching is visible, whereas a thin (≈0.5 nm) layer is observed on CdZnTe samples with 10% zinc concentration. We observed a rapid surface layer growth within five days after the sample treatment. Afterwards a semi-saturation of the layer thicknesses in all of the studied samples is visible within days. The surface layer growth rate is a valuable parameter and its knowledge gives the opportunity to properly optimize the detector fabrication and to overcome the limited lifetime of their current generation. The absolute thicknesses and the growth rate of all samples are similar within the margin of error. About 3 nm of the surface layer grew on CdTe and CdZnTe samples during one month. We investigated the leakage current decrease in the same time frame as the oxide growth. Thicker oxide layer correlates with a lower value of leakage current. We have successfully clarified the changes in the detector performance with time after its preparation. Moreover, optical properties of bulk CdTe and CdZnTe materials were deduced from ellipsometric measurements. The optical absorption edges were in agreement with theoretical predictions showing the correctness of theoretical fits and model structure.

## Disclosure statement

No potential conflict of interest was reported by the authors.

## Funding

This paper was financially supported by the Technological Agency of Czech Republic under no. TE01020445, the Czech Science Foundation under No. GAČR 15–05259S, the Grant Agency of Charles University (project No. 1054213) and student project SVV–2016–260325.
